# Giant adrenal myelolipoma: Incidentaloma with a rare incidental association

**DOI:** 10.4103/0974-7796.68865

**Published:** 2010

**Authors:** Nisar Ahmad Wani, Tasleem Kosar, Ijaz A. Rawa, Abdul Qayum

**Affiliations:** Department of Radiodiagnosis and Imaging, Sher-I-Kashmir Institute of Medical Sciences (SKIMS), Srinagar, J & K, India; 1 Department of Urology, Sher-I-Kashmir Institute of Medical Sciences (SKIMS), Srinagar, J & K, India

**Keywords:** Adrenal gland, Bochdalek hernia, multidetector-row CT, myelolipoma

## Abstract

Adrenal myelolipoma is an unusual, benign and biochemically inactive tumor that is composed of mature adipose and hematopoietic tissue. It is usually diagnosed accidentally and nowadays much more frequently because of widespread use of ultrasonography, computed tomography (CT) and magnetic resonance imaging. Adrenal myelolipoma is usually unilateral and asymptomatic, though known to be associated with obesity, hypertension, endocrinological disorders and some malignancies. We report herein two cases of right-sided giant adrenal myelolipoma diagnosed by multidetector-row CT. One patient was symptomatic because of a large mass in the right upper abdomen, which on imaging with CT was seen to be right adrenal myelolipoma. Another patient had a large left side Bochdalek hernia and right adrenal myelolipoma was incidentally discovered on CT.

## INTRODUCTION

Myelolipoma is an uncommon tumorlike lesion composed of a variable mixture of hematopoietic elements and mature fat that resembles bone marrow.[1] The term “myelolipoma” was used by Oberling in 1929,[2] though the lesion was described earlier by Gierke in 1905.[3] Myelolipoma has most often been found in adrenal gland but extra-adrenal locations are also known.[1,4] This lesion does not cause an endocrine disorder by itself and no malignant potential has been demonstrated. Adrenal myelolipomas are usually noted in late adult life and affect males and females equally.[1,4,5] With an incidence of 0.08–0.2%, myelolipomas account for 3–5% of all primary tumors of adrenals. With the widespread use of noninvasive imaging with ultrasonography (US), computed tomography (CT) and magnetic resonance imaging (MRI), incidental detection of myelolipomas is becoming more common, constituting up to 10–15% of incidental adrenal masses.[5,6] Majority of these incidentalomas are unilateral, small and asymptomatic. We report two cases of giant right adrenal myelolipoma detected with multidetector-row CT (MDCT), one in an asymptomatic patient with left side Bochdalek hernia and another in a patient with right abdominal pain.

## CASE REPORTS

### Case 1

**Figure 1:**
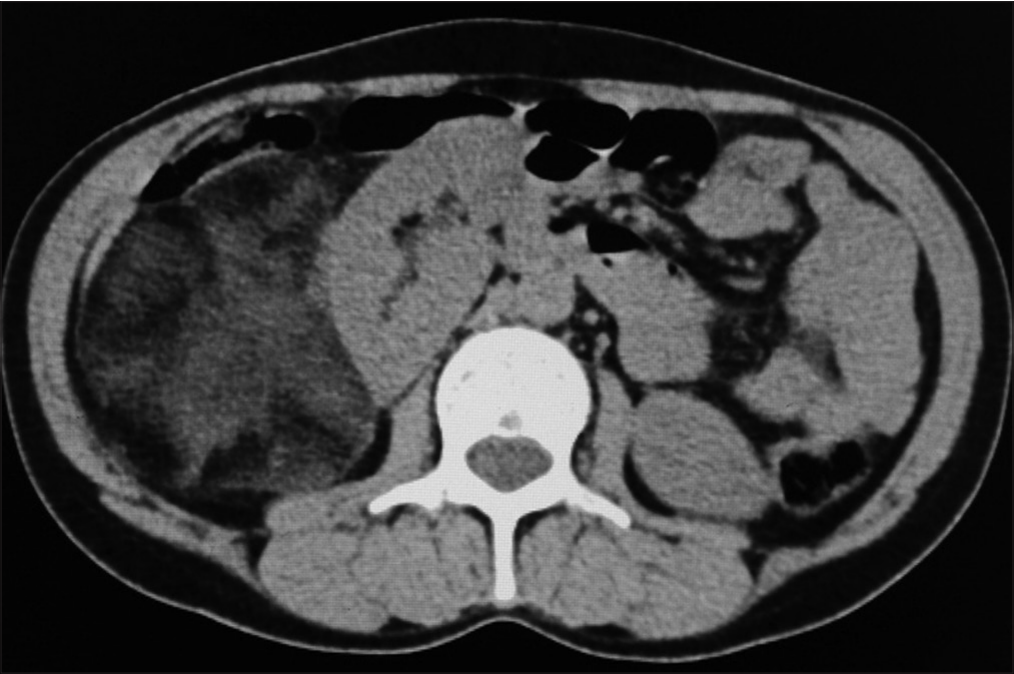
Noncontrast transverse CT image showing a large well-delineated fatty mass with some dense attenuation areas inside on the lateral aspect of right kidney which is displaced medially

**Figure 2:**
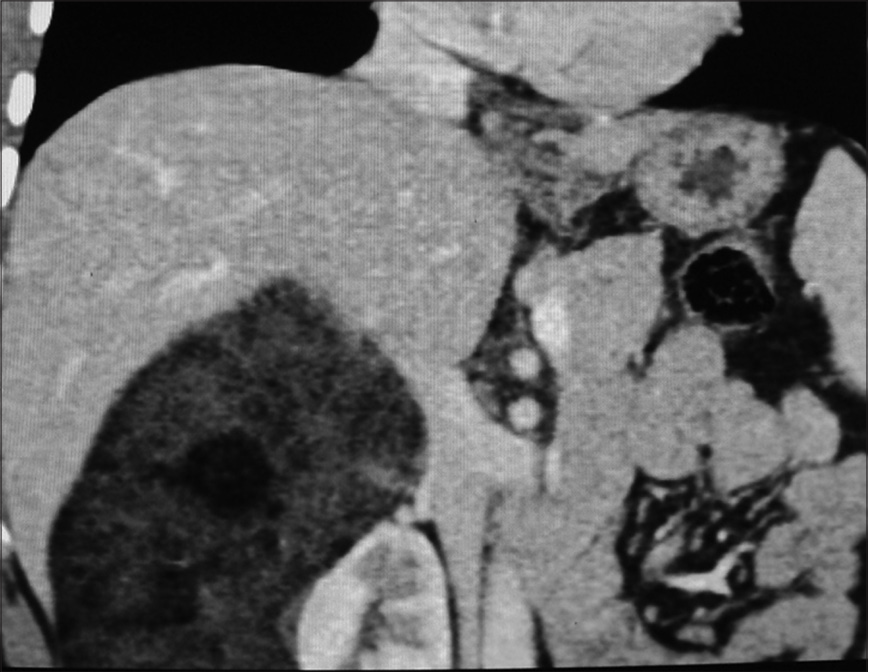
Coronal reformation contrast enhanced CT shows the fatty mass with some dense components on the superior and lateral aspects of right kidney displacing it medially

**Figure 3:**
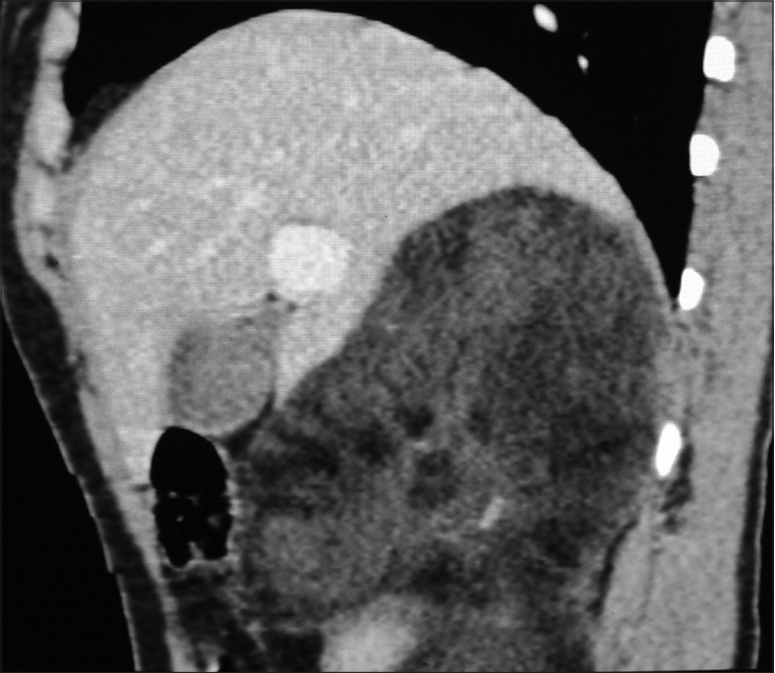
Sagittal reformation CT shows the fat density mass with some more dense components inside, interposed between liver and upper pole of right kidney

**Figure 4:**
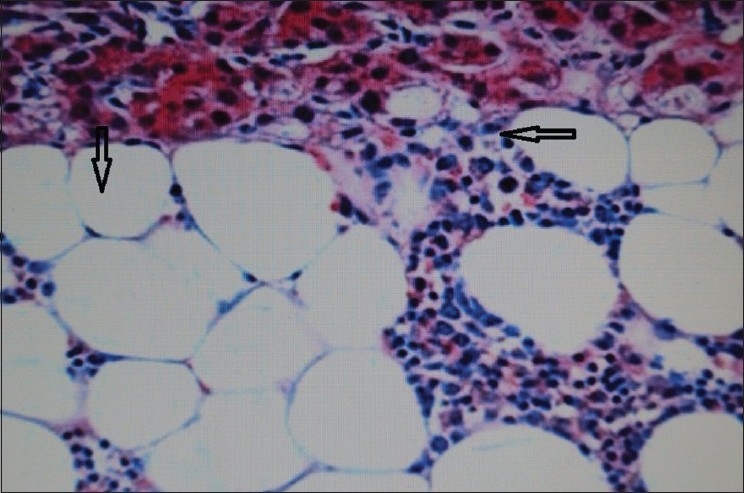
Histopathologic picture of myelolipoma showing mature fat (downward arrow) and myeloid tissue (leftward arrow)

A 52-year-old man with a history of hypertension presented with the complaint of pain in right upper abdomen for last 15 days. He was afebrile and well built. On per-abdomen examination, a bimanually palpable mass was noticed in the right lumbar region. Ultrasonography revealed a hyperechoic mass measuring 15 cm on the lateral aspect of right kidney displacing it medially. CT study of abdomen was performed with an MDCT for the evaluation of the mass in right upper abdomen. MDCT revealed a large (16 cm×12 cm) mass in the retroperitoneum on the superior and lateral aspects of right kidney which was displaced inferiorly and medially [Figures 1–3]. The mass appeared largely fatty with areas of higher attenuation inside. Density on CT varied between –90 Hounsfield units (HU) peripherally to –30 HU in the center [Figures 1–3]. Some punctate foci of calcification were also seen in the suprarenal mass. Location and attenuation of the mass on CT were suggestive of right adrenal myelolipoma. Surgery was performed through a right subcostal incision for the extraperitoneal approach of the right adrenal gland. The mass was totally dissected from the right kidney and excised. The macroscopic examination of the mass revealed a giant mass measuring 16×12×10 cm, surrounded by a thin capsule. The pathology report revealed myelolipoma of the right adrenal gland [Figure 4]. Three months after surgery, patient was pain free and no recurrent mass was seen on CT.

### Case 2

**Figure 5:**
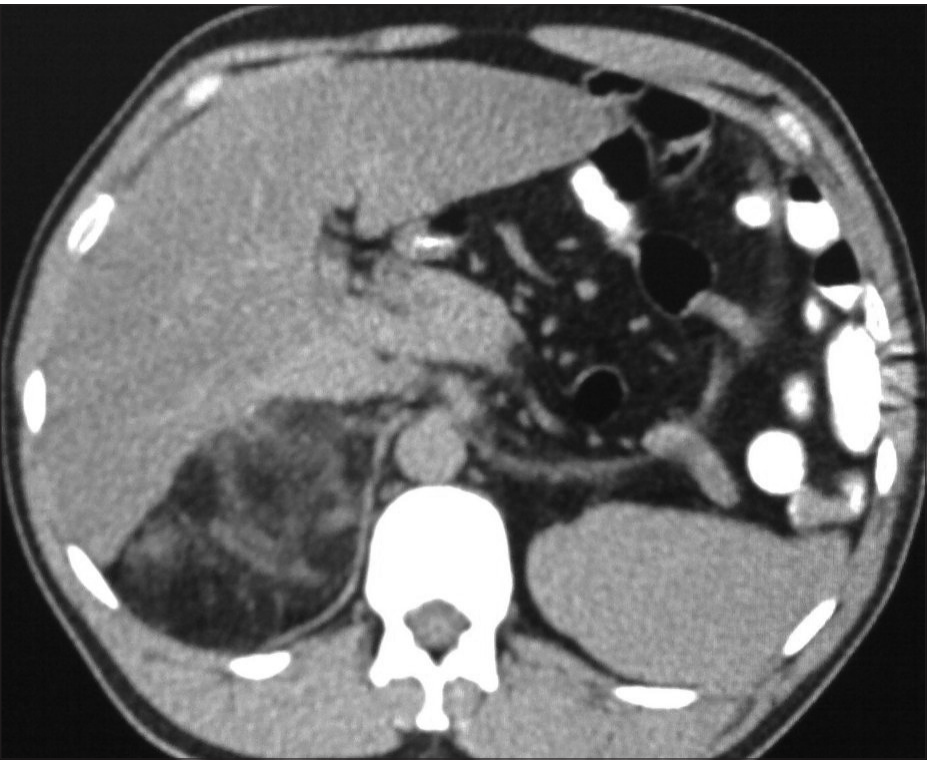
Noncontrast enhanced transverse CT image showing a large fat density mass in the right adrenal with some denser components in it; left hemidiaphragm shows posterolateral defect with luminal contrast filled stomach and small intestine and spleen posterior to it inside left hemithorax

**Figure 6:**
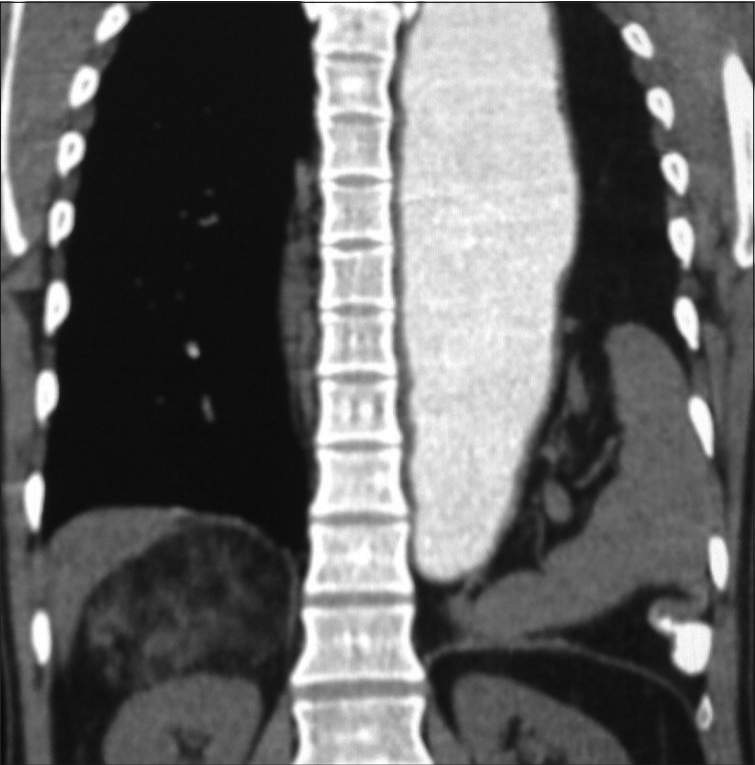
Coronal reformation CT shows the whole of stomach with luminal contrast, multiple small intestine coils, colon and spleen above left hemidiaphragm inside left hemithorax; fat attenuation mass with more dense areas inside is seen in the right adrenal gland

A 48-year-old man being evaluated for hypertension was advised a chest X-ray by the treating physician, which showed stomach bubble in the left chest. CT was performed after oral ingestion of barium to look for a diaphragmatic hernia. MDCT with coronal reformation showed a large defect in the posterolateral left hemidiaphragm with stomach, small intestine, spleen and colon inside the left hemithorax [Figures 5 and 6]. A well-defined fat attenuation mass measuring 10×8 cm was seen in the right adrenal region between liver and kidney with some higher attenuation areas within, suggesting a diagnosis of adrenal myelolipoma [Figures 5 and 6]. Patient was planned to be operated for left Bochdalek hernia first before removing the right adrenal mass.

## DISCUSSION

Adrenal myelolipomas have been somewhat of medical curiosity as they are uncommon, benign and usually asymptomatic.[6] They are composed of mature fat and hemopoietic tissues including erythroid, myeloid, megakaryocyte types and often lymphocytes. Unlike the bone marrow, myelolipoma lacks bony spicules and reticular sinusoids.[6] The most accepted theory of etiopathogenesis of adrenal myelolipoma is metaplasia of the reticuloendothelial cells of blood capillaries in the adrenal gland in response to stimuli such as necrosis, infection, stress or long-term adrenocorticotrophic hormone (ACTH) stimulation.[1,4–7]

Adrenal myelolipoma is mostly discovered on imaging for unrelated reasons and constitutes 10–15% of adrenal incidentalomas. Occasionally, patients present with abdominal pain secondary to hemorrhage, tumor necrosis or mechanical compression; other rare presenting symptoms include hematuria and abdominal mass.[1,4,5,7] Four distinct clinico-pathological patterns have been described – isolated adrenal myelolipoma, adrenal myelolipoma with hemorrhage, extra-adrenal myelolipoma and myelolipoma associated with other adrenal diseases like adrenal adenomas or endocrine disorders including Cushing’s syndrome, Conn’s syndrome and congenital adrenal hyperplasia.[1,4,5] The association of adrenal myelolipoma with obesity, hypertension, atherosclerosis, diabetes mellitus and malignancy has been described previously.[8] Both our patients had hypertension. One of them presented with abdominal pain and had palpable mass; in other patient with asymptomatic large left side Bochdalek hernia, myelolipoma was discovered incidentally. This is perhaps the first report of an incidental giant myelolipoma in a case of huge incidental Bochdalek hernia.

Bochdalek hernia is an extension of intra-abdominal contents into the chest due to posterolateral defects in diaphragm, predominant on the left side. Usually diagnosed in the first few weeks of life due to respiratory distress in neonates, Bochdalek hernias are mostly asymptomatic in adults. MDCT enhances the detection of diaphragmatic defects with its multiplanar reformations and delineates the contents which may include fat, retroperitoneal structures or intraperitoneal contents, although the latter two conditions are exceedingly rare. These hernias are incidental imaging findings in adult being evaluated for chest or abdominal pathologies.[9] We report an unusual association of an incidentaloma on the right side in the form of adrenal myelolipoma in a case of large incidental Bochdalek hernia on the left.

US, CT and MRI are effective in diagnosing more than 90% of adrenal myelolipomas based on identification of fat, with CT being the most sensitive.[5,6,10,11] US shows myelolipoma as a well-defined tumor with varying degrees of hyperechoic (fatty tissue) and hypoechoic (myeloid tissue) components. MRI of adrenal myelolipoma characteristically demonstrates a bright signal on T1-weighted and T2-weighted sequences with a decrease in signal intensity on fat suppression and opposite phase imaging, consistent with the presence of fat that is confirmatory of adrenal myelolipoma. MDCT with multiplanar reformations helps in determining the origin of tumor besides the tissue characterization and assessment of tissue planes. Myelolipomas on CT appear as well-delineated heterogenous attenuation masses with low-density (<–30 HU) fat tissue interspersed with more dense areas of myeloid tissue.[5,6,10,11] Myelolipomas can vary in size from less than 1 cm to more than 30 cm but are usually <5 cm in diameter; calcification is seen in about 27%. A fatty adrenal mass is diagnostic of myelolipoma with a possible differential diagnosis of adrenal adenoma, lipoma, angiomyolipoma and retroperitoneal sarcoma. If CT shows nonhomogenous characteristics or if the diagnosis is in doubt, an image-guided needle fine biopsy could be performed to confirm the diagnosis.[5,12]

Management of adrenal myelolipomas should be individualized. The asymptomatic small lesions less than 4 cm should be followed up with US or CT. Surgery is indicated when the patient is symptomatic, when the lesion is more than 5 cm in size due to rare chances of rupture, or if malignancy is suspected. The laparoscopic approach seems to be gaining ground for the management of adrenal tumours including myelolipomas. However, this procedure is not indicated for masses larger than 10 cm or with adhesions and infiltration of the surrounding structures.[6,13]

We conclude that MDCT helps in the preoperative diagnosis of giant adrenal myelolipoma depicting the tumor and its origin very well.
